# Identification of putative unique immunogenic ZIKV and DENV1-4 peptides for diagnostic cellular based tests

**DOI:** 10.1038/s41598-017-05980-z

**Published:** 2017-07-24

**Authors:** Aaron L. Oom, Davey Smith, Kevan Akrami

**Affiliations:** University of California, San Diego, Department of Infectious Disease, 9500 Gilman Drive #0711, La Jolla, CA 92093-0711 USA

## Abstract

Since the re-emergence of Zika virus in 2014 and subsequent association with microcephaly, much work has focused on the development of a vaccine to halt its spread throughout the world. The mosquito vector that transmits this virus is widespread and responsible for the spread of other arboviridae including Dengue. Current diagnostic methods rely on serologic testing that are complicated by cross reactivity and therefore unable to distinguish Zika from Dengue infection in the absence of virus isolation. We performed an *in silico* analysis to identify potential epitopes that may stimulate a unique T-lymphocyte response to distinguish prior infection with Zika or Dengue. From this analysis, we not only identified epitopes unique to Zika and Dengue, but also identified epitopes unique to each Dengue serotype. These peptides contribute to a pool of peptides identified for vaccine development that can be tested *in vitro* to confirm immunogenicity, absence of homology and global population coverage. The current lack of accurate diagnostic testing hampers our ability to understand the scope of the epidemic, implications for vaccine implementation and complications related to monoinfection and co-infection with these two closely related viruses.

## Introduction

Zika virus (ZIKV) is a member of the Flavivirus genus related to Dengue virus (DENV) and was first isolated in 1947 from a Rhesus macaque in the Zika forest of Uganda^1^. ZIKV infection commonly presents sub-clinically with only about 20% of infected acknowledging symptoms, and when symptoms are present they are usually fever, rash, and constitutional symptoms similar to those seen during DENV infections. These acute clinical similarities are confounded by a high coincidence of geographical ranges of the two infections^2^ thanks to a shared mosquito vector, *Aedes aegypti* ^3^.

Efforts to distinguish ZIKV and DENV infections, especially after the acute phase, are complicated by the large degree of genetic similarity and serologic responses between the two viruses^4–9^. Many serologic tests are available for ZIKV and DENV, including IgM antibody capture-ELISA (MAC-ELISA) and Plaque Reduction Neutralization Test (PRNT), but these platforms often cannot reliably distinguish between the two viruses due to antibody cross-reactivity and low sensitivity^10^. Cross-reactivity has hindered accurate epidemiological tracking of ZIKV. A commercially available ELISPOT for DENV has demonstrated increased sensitivity when compared to serologic or antigen assays though did not assess for potential cross-reactivity with ZIKV^11^. This uncertainty makes clinical and epidemiological studies of ZIKV and DENV in human populations difficult to understand. Prior work with H1N1 [ref. 12], Hepatitis B^13^, and Mycobacterial infections^14, 15^ has demonstrated the utility of epitope recognition in the development of novel diagnostics and vaccines. Therefore, we propose that detection of a specific lingering cytotoxic T-lymphocyte (CTL) memory response against Zika and/or Dengue, unlike antibody responses, would allow these infections to be distinguished. To this end we performed an *in silico* analysis of ZIKV non-structural protein 1 (NS1) and DENV non-structural protein 2a (NS2a) to determine virus-specific epitopes that could be used in the development of novel diagnostic tests to distinguish these infections. These proteins were chosen as previous data has suggested that DENV NS2a will have minimal ZIKV CTL cross reactivity^16, 17^ and that ZIKV NS1 is largely ZIKV specific^10, 18, 19^.

## Results

Assays that can detect human CTL immune response to pathogens require specificity to unique pathogen proteins that are presented to CTLs *in vivo*. To be useful across the entire circulating pathogen population, these assays will need to use relatively conserved peptides in the pathogen of interest. This may be especially difficult for viruses, which cover a large range of genetic diversity. To tackle the challenge of creating a CTL assay that can distinguish ZIKV from DENV, we first collected all protein sequences of these viruses that are likely to be presented to the human CTL immune system during acute infection. We focused on NS1 for ZIKV and NS2a for DENV because these regions were found in previous studies to likely be immunogenic and unique regions with little cross-reactivity between ZIKV and DENV CTL responses^10, 16–19^. We then analyzed sequences for conservation across all known isolates for ZIKV and all DENV sequences available since 2010, which would increase the fidelity of any potential diagnostic assay. We used the following criteria to evaluate which peptides were most likely to generate a pathogen specific CTL response across the human population according to predicted ability to: (1) bind to 3 or more MHC-Class I alleles, (2) bind to 2 or more MHC-II alleles, (3) have a probability score of at least 0.8 (near match or perfect match) for natural processing of peptides and subsequent MHC-I binding in the cell, (4) be immunogenic according to which amino acids are present at positions 4–6 and the presence of large and aromatic side chains in 9-mers, (5) have population coverage of at least 60%, (6) dock in the binding groove of the MHC molecule, and (7) be specific to DENV or ZIKV (i.e. absence of homology). While not all predicted epitopes met the above criteria, they may be used in combination to form a ZIKV and DENV peptide pool to be tested for CTL response *in vitro*.

For the ZIKV NS1 protein sequence, we used a 2015 Brazilian isolate retrieved from the Protein Database (5GS6 [ref. 20]). To ensure that this sequence was representative of all known isolates, the sequence was inputted in a BLASTp analysis against all available full-length ZIKV protein sequences from the NCBI’s Virus Variation Zika Database (182 sequences)^21^; both African and Asian clade samples were present to account for any potential clade differences within NS1. Every subject sequence covered residues 7 to 357 (C-terminus) and differed by only 1 residue at most against all ZIKV NS1 regions.

For the DENV NS2a, a consensus amino acid sequence from all 4 serotypes was constructed using DENV NS2a sequences collected between 2010 and 2016 from human hosts (563 sequences) obtained from the NIAID Virus Pathogen Database and Analysis Resource (ViPR)^22^. When analyzing this sequence using BLASTp, we found that the consensus sequence was biased towards DENV1 NS2a with 74–82% sequence similarity to DENV1 NS2a. When compared with the other serotypes (DENV2-4), sequence similarity ranged from 45–58%. The higher prevalence of DENV1 sequences in viral databases over other serotypes suggests that DENV1 may be over-represented when constructing a consensus sequence. Therefore, we chose to examine each DENV serotype in addition to the consensus sequence.

How a cell infected with either ZIKV or DENV processes peptides prior to the presentation and binding of peptides to MHC molecules is important to determine the potential for an epitope to interact with the CTL receptor. Following identification of the consensus amino acid sequences for ZIKV and DENV (all 4 serotypes and serotype specific), we next evaluated each of the peptides for their ability to undergo natural processing and bind to MHC I alleles. We then compared the potential immunogenicity of candidate peptides based on their biochemical structure (i.e. presence of aromatic rings)^23^ as assessed by the IEDB immunogenicity tool.

### MHC Peptide Processing and Binding

**Figure 1:**
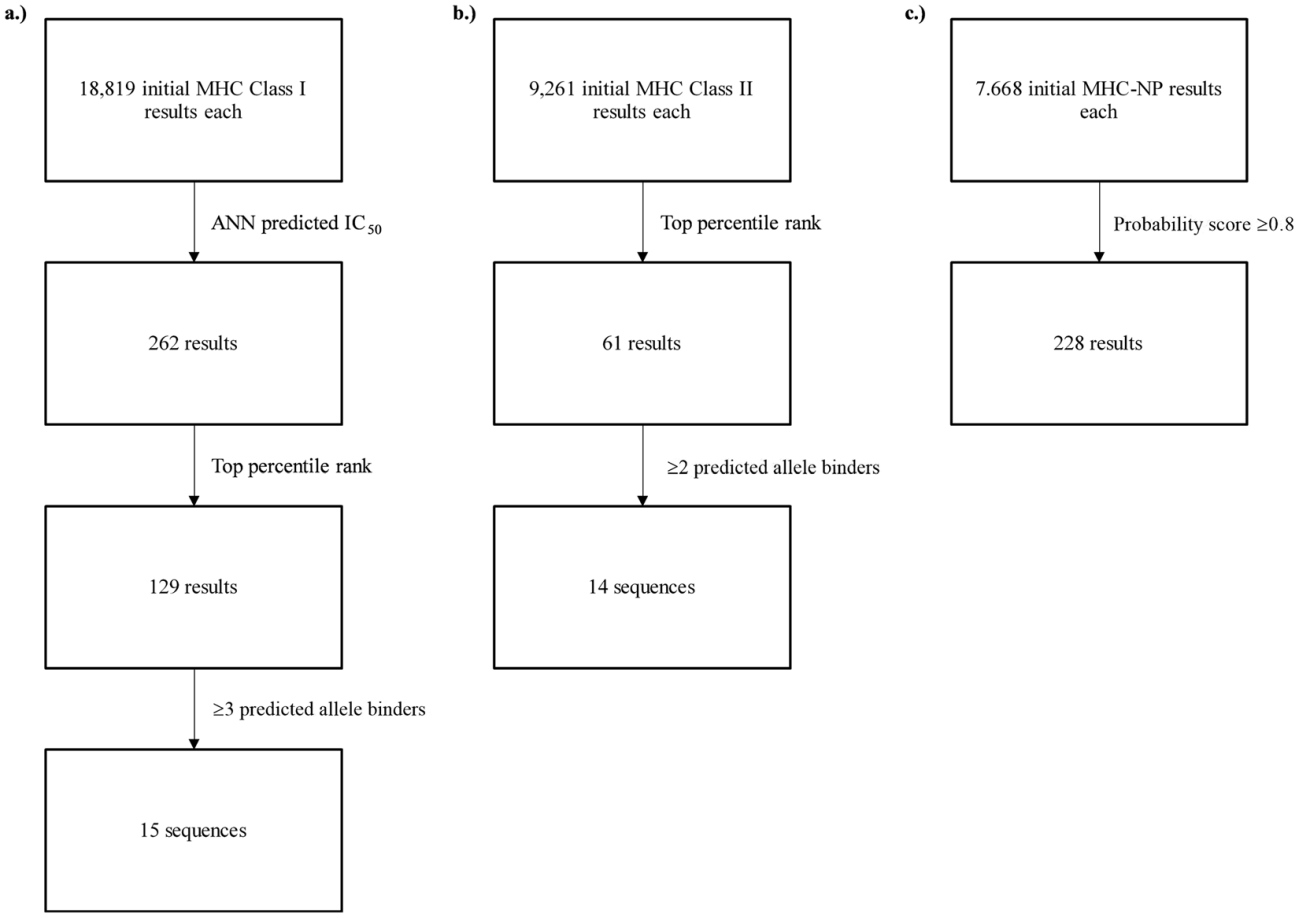
Workflow for ZIKV peptide antigenicity predictions. Briefly, chain A of 5GS6 from the Protein Database was submitted to the IEDB MHC class I and II binding tools and MHC-NP tool. (**a**) Class I results were first filtered by ANN predicted IC_50_ levels as recommended in Paul *et al*.^39^ followed by filtering for those results in the top 1 percentile rank. Subsequent results were then sorted by sequence and those predicted to bind ≥3 class I alleles were chosen for further analysis. (**b**) Class II results were first filtered for those results in the top 1 percentile rank and then sorted by sequence with those predicted to bind ≥2 class II alleles chosen for further analysis. (**c**) MHC-NP initial results were filtered for those hits with a probability score of ≥0.8. Cross-referencing of class II and MHC-NP data against class I data was performed to generate putative peptides (Table 1).

First, we evaluated the ability of potential epitopes in the ZIKV consensus strain 5GS6 NS1 to bind to MHC class I and II following MHC natural processing (Fig. 1a–c and Table 1). This provided us with 2 potential ZIKV peptides to then evaluate for immunogenicity, population coverage, docking and cross-reactivity with other flaviviridae including DENV (Table 1).

**Table 1:** Predicted ZIKV NS1 epitopes from consensus ZIKV NS1 sequence, Dar *et al*., and Dikhit *et al*.

NS1 Residues	Sequence	HLA Class	*Flaviviridae* Homology (E-value <1)?	World Coverage	South America Coverage	Immunogenicity
16–24/25	KETRCGTGV/KETRCGTGVF*	I	DENV2-4 NS1	20.88%	10.34%	0.09664/0.13444
125–133	KSYFVRAAK*	I	None	38.48%	28.66%	0.2614
158–167	FLVEDHGFGV**^@^	I	DENV1-4 NS1	41.35%	21.85%	0.31775
166–175	GVFHTSVWLK*	I & II	DENV1&3-4 Peptidase S7, NHUV glycoprot.	69.67%	78.11%	0.16657
169–177	HTSVWLKVR**	I & II	DENV1-3 NS1	58.80%	79.86%	0.03522
*1059*	KGPWHSEEL	I	None	Dar *et al*.^26^ Polyprotein position used in residue numbering *All peptides bind* >*3 alleles*		
*878*	VQLTVVVGS	II	None			
*878*	VQLTVVVGSVKNPM	II	None			
*965*	VREDYSLEC	II	None			
*982*	VKGKEAVHS	II	None			
*1004*	WRLKRAHLI	II	None			
*1045*	LSHHNTREG	II	None			
*1124*	WYGMEIRPR	II	WNV NS1			
80–88	ILEENGVQL	I	None	Dikhit *et al*.^25^ NS1 protein position used in residue numbering *All peptides bind* >*3 alleles*		
87–95	QLTVVVGSV	I	None			
364–372	SLGVLVILL	I	None			
366–374	GVLVILLMV	I	None			
370–378	ILLMVQEGL	I	None			

*Exact MHC-NP Matches.

**Near MHC-NP Matches.

^@^Due to high immunogenicity and DENV1-4 homology, this peptide may serve as a positive control for DENV/ZIKV infection.

Immunogenicity >0 is the threshold for predicted immunogenicity.

**Figure 2:**
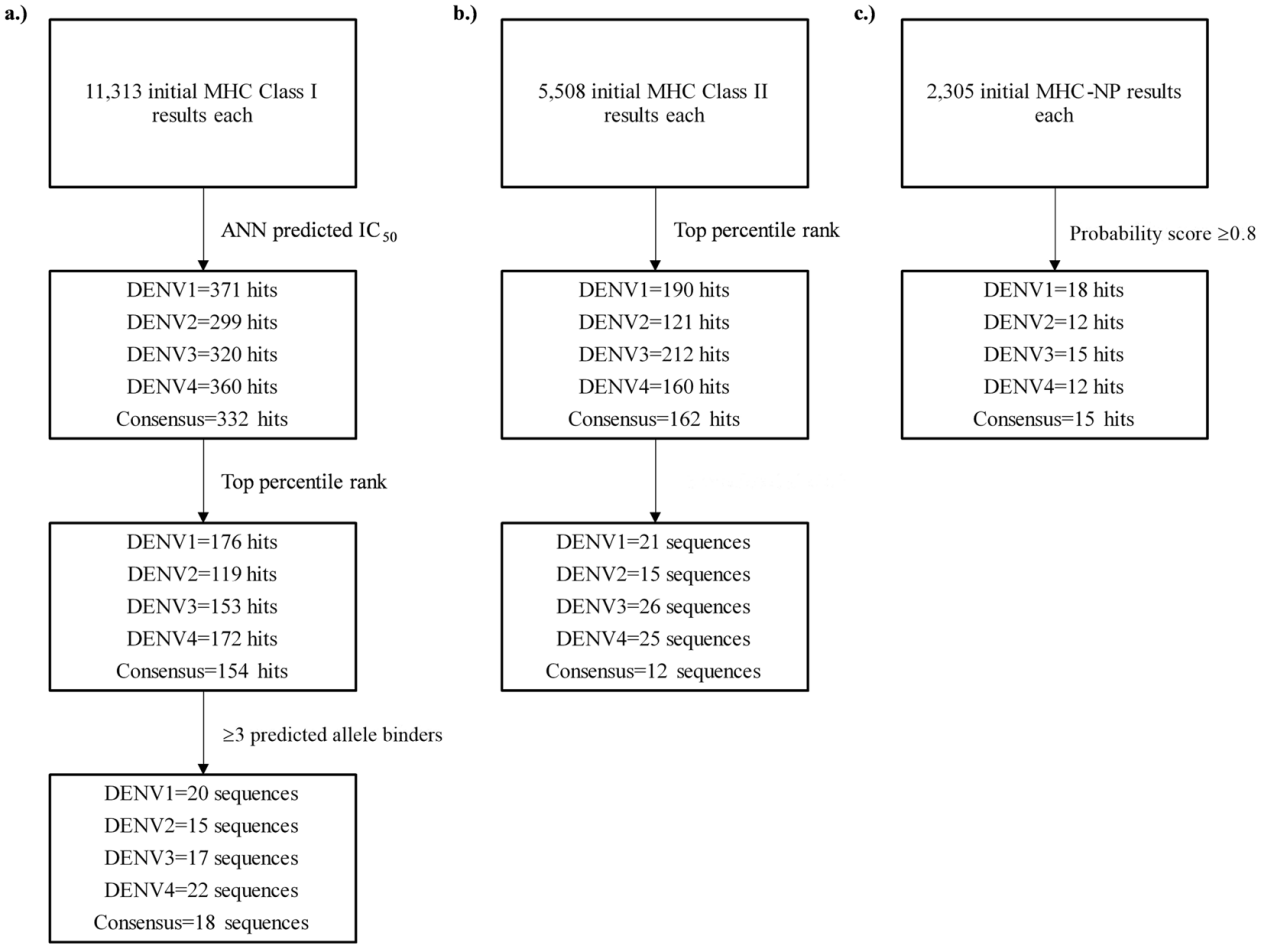
Workflow for DENV peptide antigenicity predictions. Briefly, each serotype NS2a consensus sequence as well as the DENV NS2a consensus sequence were submitted to the IEDB MHC class I and II binding tools and MHC-NP tool. (**a**) Class I results were first filtered by ANN predicted IC_50_ levels as recommended in Paul *et al*.^39^ followed by filtering for those results in the top 1 percentile rank. Subsequent results were then sorted by sequence and those predicted to bind ≥3 class I alleles were chosen for further analysis. (**b**) Class II results were first filtered for those results in the top 1 percentile rank and then sorted by sequence with those predicted to bind ≥3 class II alleles chosen for further analysis. (**c**) MHC-NP initial results were filtered for those hits with a probability score of ≥0.8. Cross-referencing of class II and MHC-NP data against class I data was performed to generate putative peptides (Table 2).

Second, we evaluated the consensus sequence of DENV NS2a and found three peptides that were predicted to bind class I and II molecules though were not predicted to be naturally processed (Fig. 2a–c and Table 2). Given the lack of an epitope in the DENV NS2a consensus sequence that fulfilled the initial binding and processing criteria, we focused on the identification of serotype specific peptides that could be used to identify prior DENV infection in a serotype specific way (Fig. 2a–c). Serotype specific identification in clinical practice is rarely performed as it requires isolation and molecular typing of DENV during acute infection. Therefore, we chose to identify potential peptides that may distinguish the serotype of prior infection. For DENV1, a single potential peptide, MLMTGTLAV/MLMTGTLAVF, was predicted to meet our MHC class I binding and natural processing criteria. For DENV2, we found one epitope, ILLVAVSFV, that fulfilled all criteria and another that met natural processing and MHC-I binding but not MHC-II binding, TMTDDIGMGV. For DENV3 and DENV4, we found 3 (HMIAGVFFTF, MIAGVFFTF/MIAGVFFTFV, LLLSGQITW) and 2 (KMSPGYVLGV, STMSLVMAW/STMSLVMAWR) epitopes respectively that met our binding and processing criteria.

**Table 2:** Predicted DENV NS2a epitopes for DENV1-4 and consensus DENV NS2a sequence.

Serotype	Residues	Sequence	MHC II Binding?	ZIKV Homology (E-value <1)?	Other DENV Homology (E-value <1)?	World Coverage	S. America Coverage	Immunogenicity
1	31–39/40	MLMTGTLAV/MLMTGTLAVF**	Yes, <3 alleles	None	None	86.75%	67.85%	0.0651/0.09956
2	33–41	ILLVAVSFV**	Yes	None	None	72.68%	57.84%	0.04368
	63–72	TMTDDIGMGV*	No	None	None	41.35%	21.85%	0.08962
3	31–40	HMIAGVFFTF*	Yes	None	None	84.20%	83.18%	0.38745
	32–40/41	MIAGVFFTF/MIAGVFFTFV*	Yes	None	None	94.28%	89.85%	0.31868/0.41236
4	81–90	KMSPGYVLGV*	Yes	None	None	51.64%	30.96%	0.01384
Consensus	15–23/24	MAIFIEEVM/MAIFIEEVMR	Yes	None		44.73%	31.63%	0.49347/0.41459
	42–50/51	LLIMGQLTW/LLIMGQLTWR*	Yes, <3 alleles	NS4b		66.08%	78.82%	−0.19622/−0.06156
	86–94/95	MFAVGLLLR/MFAVGLLLRK	Yes	None		66.08%	78.82%	0.06096/0.08688
	132–140	MMLKLVTNF*	Yes, <3 alleles	None		34.12%	14.89%	−0.16356
	143–152	YQLWTTLLSL	Yes	None		78.77%	86.15%	0.17843
	170–178	MVLAVVSLF**	Yes, <3 alleles	None		43.07%	27.68%	−0.03127

*Exact MHC-NP match.

**Near MHC-NP match.

Immunogenicity >0 is the threshold for predicted immunogenicity.

**Table 3:** Proposed diagnostic kit peptide summary.

Peptide	Residues	Sequence	HLA Class	*Flaviviridae* Homology (E-value <1)?	World Coverage	S. America Coverage	Immunogenicity
ZIKV/DENV control	125–133	KSYFVRAAK*	I	None	38.48%	28.66%	0.2614
ZIKV	158–167	FLVEDHGFGV**	I	DENV1-4 NS1	41.35%	21.85%	0.31775
ZIKV	166–175	GVFHTSVWLK*	I & II	DENV1&3-4 Peptidase S7, NHUV glycoprot.	69.67%	78.11%	0.16657
DENV1	31–39/40	MLMTGTLAV/MLMTGTLAVF**	I & II	None	86.75%	67.85%	0.0651/0.09956
DENV2	33–41	ILLVAVSFV**	I & II	None	72.68%	57.84%	0.04368
DENV3	32–40/41	MIAGVFFTF/MIAGVFFTFV*	I & II	None	94.28%	89.85%	0.31868/0.41236
DENV4	81–90	KMSPGYVLGV*	I & II	None	51.64%	30.96%	0.01384
DENV Control	86–94/95	MFAVGLLLR/MFAVGLLLRK	I & II	None	66.08%	78.82%	0.06096/0.08688
	143–152	YQLWTTLLSL	I & II	None	78.77%	86.15%	0.17843

*Exact MHC-NP match.

**Near MHC-NP match.

Immunogenicity >0 is the threshold for predicted immunogenicity.

### Immunogenicity and Homology

We then tested the epitopes for ZIKV and DENV identified above for sequence homology using BLASTp and immunogenicity using the IEDB immunogenicity tool, which has been validated for 9-mers that bind MHC-I alleles^23^. For ZIKV NS1 we found one peptide, GVFHTSVWLK, predicted to be a MHC class I and II binder, naturally processed, and immunogenic. Following BLASTp search this epitope demonstrated homology with DENV serotypes 1, 3, and 4 (Table 1). From this, it became apparent that satisfaction of all of our criteria above for a unique ZIKV peptide without homology with DENV will be difficult to identify. Therefore, we repeated the binding analysis for ZIKV NS1 (requiring only MHC I binding of 3 or greater alleles and at least 0.8 probability of natural processing without MHC II binding), immunogenicity and homology testing. From this analysis, the epitope, KSYFVRAAK, appeared to be the best candidate peptide for eliciting a CTL response specific to ZIKV infections and distinct from DENV infections. Our revised search criteria also yielded the peptide, FLVEDHGFGV, with immunogenicity and homology to a sequence from all 4 DENV serotypes in their NS1 region. Taken together, it is likely that this peptide may be used in a CTL based assay to identify those infected with either ZIKV and/or DENV.

We then evaluated the DENV NS2a potential binders for immunogenicity and homology with ZIKV. For the DENV NS2a consensus sequence, as above, we could not identify peptides that satisfied all 3 binding criteria. Therefore, we identified three peptides predicted to bind class I and II molecules but were not MHC-NP matches for further analysis. Of these three peptides, YQLWTTLLSL demonstrated high predicted immunogenicity and no significant homology detected against ZIKV sequences, suggesting a potential role as an epitope to detect prior infection with any of the 4 DENV serotypes. For the DENV1 NS2a predicted binding epitope, we found a positive immunogenicity score (Table 2) with no significant homology to ZIKV or DENV2-4 sequences, which suggests that this may be a unique peptide able to identify prior infection with DENV1. For DENV2, both sequences from the binding analysis were predicted to have positive immunogenicity scores and no significant homology to ZIKV or DENV1,3-4 sequences. For DENV3, of the three matches, two sequences were predicted to have highly positive immunogenicity scores. Interestingly, both of the hits (HMIAGVFFTF and MIAGVFFTF/MIAGVFFTFV) start around residues 31–33 of the NS2a protein, a small sequence starting range that appears to be antigenic in DENV1 and DENV2 as well, but not in DENV4 (Table 2). MIAGVFFTF/MIAGVFFTFV showed no significant homology to ZIKV or DENV1-2,4 sequences. For DENV4, only one of the two predicted binders, KMSPGYVLGV, was predicted to have a positive immunogenicity score (Table 2). Further, KMSPGYVLGV showed no significant homology to ZIKV or DENV1-3 sequences. In summary, our analysis revealed peptides for each DENV serotype that are immunogenic and unique for each serotype without apparent homology to ZIKV or other flaviviridae. Further, the ZIKV analysis revealed not only several immunogenic peptides that may be capable of distinguishing prior ZIKV from DENV infection, but also one immunogenic peptide that demonstrates shared homology between ZIKV and DENV NS1, which may trigger a CTL response in samples from those previously infected with ZIKV and/or DENV.

### Predicted Population Coverage

Given the global scope of populations at risk for these arboviral infections, our epitopes must be capable of binding to a broad range of HLA alleles and therefore offer wide coverage of the human population. Thus, we evaluated the population coverage for our promising ZIKV and DENV peptides described above using the IEDB coverage tool (Tables 1 and 2). It appeared that ZIKV peptide, KSYFVRAAK, might have quite low coverage globally (38.5%) and in South America (28.7%), which suggests that multiple ZIKV peptides may be needed in the potential assay for optimal population coverage. For the DENV NS2a consensus sequence, YQLWTTLLSL was predicted to have world coverage of 78.77% and South American coverage of 86.15%. When evaluated by serotype, the highest predicted South American population coverage was 67.85% (DENV1), 57.84% (DENV2), 89.85% (DENV3), and 30.96% (DENV4). The highest predicted global coverage for each serotype was 86.75% (DENV1), 72.68% (DENV2), 94.28% (DENV3), and 51.64% (DENV4).

### Docking Analysis

**Figure 3:**
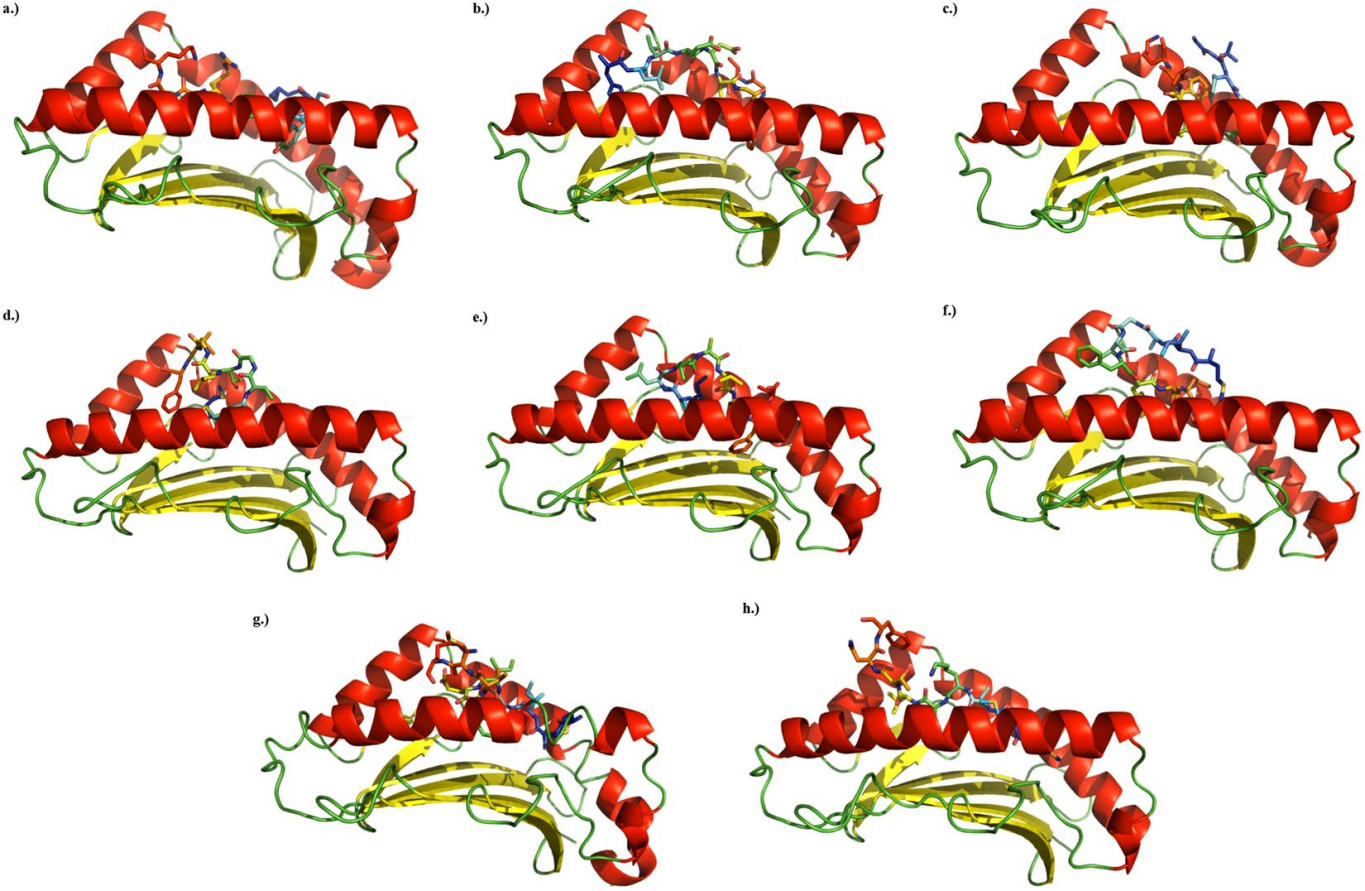
Top PatchDock predictions of epitope binding to MHC class I alleles as rendered by PyMOL Molecular Graphics System. Epitopes are shown as sticks with rainbow coloring (blue to red, N-terminus to C-terminus). HLA allele binding grooves are shown as cartoon with β-sheets in yellow, α-helices in red, and loops in green. DENV4 peptide KMSPGYVLGV did not bind within the binding groove of HLA-A*02:01 in any of the top ten PatchDock predictions. (**a**) ZIKV peptide KSYFVRAAK bound to HLA-A*03:01. (**b**) ZIKV peptide FLVEDHGFGV bound to HLA-A*02:01. (**c**) ZIKV peptide GVFHTSVWLK bound to HLA-A*03:01. (**d**) DENV1 peptide MLMTGTLAVF bound to HLA-A*02:01. (**e**) DENV2 peptide ILLVAVSFV bound to HLA-A*02:01. (**f**) DENV3 peptide MIAGVFFTFV bound to HLA-A*02:01. (**g**) DENV peptide MAIFIEEVMR bound to HLA-B*35:01. (**h**) DENV peptide MMLKLVTNF bound to HLA-B*15:01.

To further assess whether the identified epitopes would bind to HLA molecule grooves and thereby be presented to CTLs, we performed docking simulations of our predicted peptides. Docking analysis was performed for ZIKV epitopes (Fig. 3a–c) and DENV epitopes (Fig. 3d–h) to determine whether they are predicted to dock within the binding groove of HLA alleles. Both KSYFVRAAK (Fig. 3a) and FLVEDHGFGV (Fig. 3b) displayed typical or near typical binding, with FLVEDHGFGV predicted to have the most prominent arching, as has been previously described in MHC class I binding^24^. This arching leads to exposure of the internal residues, crucial for MHC class I immunogenic responses^23^, which may explain the high predicted immunogenicity of FLVEDHGFGV (Table 1). Conversely, the presentation of the terminal residues of GVFHTSVWLK (Fig. 3b) may explain its poor predicted immunogenicity (Table 1). We then evaluated whether the following predicted DENV epitopes were capable of docking within the binding groove of HLA alleles: MLMTGTLAVF, ILLVAVSFV, MIAGVFFTFV, MAIFIEEVMR, and MMLKLVTNF (Fig. 3d–h). Similar to the ZIKV predictions, MIAGVFFTFV (Fig. 3f) and MAIFIEEVMR (Fig. 3g) appeared to best display internal residues integral to a class I immunogenic response, and correlate with high predicted immunogenicity (Table 2). The presentation of the C-terminal end of MMLKLVTNF (Fig. 3h) may then explain its poor predicted immunogenicity (Table 2). The average predicted immunogenicity of the remaining Dengue peptides may be due to the varying degrees of presentation of internal residues (Fig. 3d and e). From the PatchDock analysis of the DENV4 peptide KMSPGYVLGV, there were no top results that predicted binding and/or interaction of the peptide within the HLA-A*02:01 binding groove. Docking only appeared at locations on the molecule outside of the binding groove.

## Discussion

Current efforts to identify unique ZIKV epitopes for diagnostic and vaccine testing purposes have done little to consider cross-reactivity with DENV. It is clear, however, that distinguishing DENV and ZIKV past infections would be important for research purposes to better understand the long-term consequences of each infection and co-infection. Definitive associations may be difficult in the absence of a method to properly differentiate and diagnose the two infections. Ideally, a panel of peptides can be used in CTL assays that can distinguish prior ZIKV and DENV infection (including which serotype). Our *in silico* analysis identified several epitopes that may help distinguish prior ZIKV infection from DENV by measurement of lymphocyte activation in an ELISPOT assay. Moving forward, all identified peptides (Tables 1 and 2) will need to be tested *in vitro* to confirm whether these predicted peptides can stimulate a CTL response that is specific to each virus (ZIKV and DENV1-4).

One potential limitation of our peptide pool is relatively low predicted population coverage for ZIKV peptides. Therefore, a single assay with a combination of peptides will be needed to adequately cover the broad population at risk for ZIKV infection. To this end, we have included peptides proposed by Dikhit *et al*.^25^ and Dar *et al*.^26^ from similar *in silico* analyses (Table 1) that may be included in a peptide pool to diagnose prior ZIKV infections. These studies, while similar in their approach to the one used here, have a major limitation in their focus on vaccine development that did not consider cross-reactive CTL response with Dengue, which we have addressed (Table 1). A combination of the peptides proposed here—FLVEDHGFGV (ZIKV/DENV control), KSYFVRAAK and GVFHTSVWLK (ZIKV), MLMTGTLAVF (DENV1), ILLVAVSFV (DENV2), MIAGVFFTFV (DENV3), KMSPGYVLGV (DENV4), and MFAVGLLLRK and YQLWTTLLSL (DENV control)—in conjunction with those of others may greatly increase population coverage. Additionally, peptides predicted here to have low immunogenicity (for vaccine purposes) may have a role in a diagnostic setting as a higher concentration of peptide in the assay may overcome low immunogenicity (Table 3). This also speaks to the power of IEDB in peptide discovery for diagnostic purposes as well as vaccine purposes; other services such as Predivac^27^ are more focused on vaccine generation and have a smaller range of capabilities and freedoms, typically coupling together analyses that IEDB handles independently of one another.

Our predicted peptides also focused on identifying peptides unique to each serotype of DENV, which has not been previously evaluated for diagnostic purposes. The ability to distinguish DENV serotype will be important for prospective studies as it is clear that certain serotypes and the sequence of infection with different serotypes have been more clearly associated with the development of Antibody Dependent Enhancement (ADE)^28^. The serotype specific analysis of DENV peptides included here may also contribute to prospective studies to determine whether there are clinically significant ADE-mediated effects between individual DENV serotypes and ZIKV, as has been demonstrated *in vitro* ^6, 7^ and recently in patient plasma samples and mice^29^.

Interestingly, when we analyzed all the potential peptides for the diagnostic test using the BLASTp, we found that the only peptide with sequence homology to other *Flaviviridae* family members was the ZIKV peptide GVFHTSVWLK, and it was an exact match to the Nhumirim virus (Table 1). However, only the original discoverers have sequenced the virus, collected in 2010 from Brazil, leaving open questions of how representative this particular sequence is of circulating Nhumirim virus^30^.

The inclusion of molecular structures here offers one potential explanation for the predicted immunogenicity of each peptide. Those peptides that most closely resemble the expected binding of the C- and N-terminal residues in the binding groove and the protruding arch-like presentation of internal residues^24^ are also those that are predicted to have the highest immunogenicity. These models, while potentially illuminating, have their limits. Even when limiting the HLA allele to only the binding groove as the receptor rather than the full molecule, docking predictions still have varying success in placing the peptide within the binding groove rather than adjacent to it. Whether this is a reflection on the IEDB binding prediction or on the PatchDock model itself is not clear. Validation of both IEDB and PatchDock results will require *in vitro* testing to determine CTL responsiveness to each peptide as well as to evaluate for cross-reactivity.

In areas endemic to ZIKV and DENV, Chikungunya virus has emerged as yet another virus often transmitted through *A*. *aegypti* that produces clinical symptoms similar to DENV and ZIKV infection^31^. If the current peptides are confirmed to generate specific ZIKV or DENV cytokine release and lead to a diagnostic platform, people with congruent symptoms but with negative results on this test would need to be screened for CHIKV infection. Importantly, while clinical similarities are manifest, CHIKV is of an entirely separate viral family altogether—*Togaviridae*—and molecular misdiagnosis is unlikely. In contrast to the serologic cross reactivity apparent between the flaviviridae, CHIKV triggers an immunologic response that can be distinguished from ZIKV or DENV with serological testing alone.

Here, we have demonstrated the utility of *in silico* analysis to identify a pool of peptides that may be important in the development of a CTL based test to diagnose prior infection with either DENV (including individual serotype) or ZIKV. This diagnostic tool may have implications for Dengue and Zika vaccine implementation and risk of ADE^32, 33^, women of child bearing age regarding the risk of microcephalus and identification of any long-term complications related to co-infection. Such a test will require rigorous evaluation in a clinical setting to determine efficacy and epidemiological power.

## Methods

### ZIKV NS1 Protein Sequence

The ZIKV NS1 protein sequence used here is a 2015 Brazilian isolate and was retrieved from the Protein Database (5GS6 [ref. 20]).

### DENV1-4 NS2a Protein Sequences

For DENV, a consensus amino acid sequence of NS2a from all 4 serotypes was constructed on Geneious version 10.0.6 [ref. 34] at a 25% threshold using DENV NS2a sequences. DENV NS2a sequences collected between 2010 and 2016 from human hosts (563 sequences) were obtained from and aligned by the NIAID Virus Pathogen Database and Analysis Resource (ViPR)^22^ through the web site at http://www.viprbrc.org/. We limited our search from 2010 to 2016, as this represents the consensus sequence of the most likely circulating strains of DENV. We obtained and aligned individual serotype consensus sequences through ViPR followed by generation of a consensus sequence for each serotype at a 50% threshold using Geneious version 10.0.6.

### MHC Class I and II Binding

Potential unique epitopes that bind to MHC Class I and II were identified using the Immune Epitope Database and Analysis Resource (IEDB) (http://www.iedb.org/) [ref. 35]. IEDB hosts a suite of analysis tools for epitope prediction including MHC class I and II binding predictive analysis through biophysical comparison, rather than sequence homology, of known class I and II epitopes to potential epitopes^36–38^. The MHC class I binding tool has been well-characterized by the IEDB group with defined IC_50_ thresholds as determined by the Artificial Neural Network method (ANN) for many of the most common human HLA class I alleles^39^. Chain A of 5GS6 and the generated DENV NS2a consensus sequences were tested against the MHC class I and II reference allele sets that are designed to cover ~97% and 99% of the world population, respectively^40, 41^ (Supplemental Tables S1 and S2). The approach for ZIKV and DENV peptide MHC class I and II binding are detailed as previously described (Figs 1a,b and 2a,b).

### MHC Natural Processing

The IEDB MHC Natural Processing (MHC-NP) tool predicts naturally processed epitopes based on physiochemical properties and residue position comparisons to known epitopes^42^. Chain A of 5GS6 and the generated DENV NS2a consensus sequences were submitted to the MHC-NP tool with default parameters against HLA-A*02:01, HLA-B*07:02, HLA-B*35:01, HLA-B*44:03, HLA-B*53:01, and HLA-B*57:01 for predicted 9-mers and 10-mers. Results were filtered for hits with a probability score of 0.8 or greater and cross referenced against MHC I binding data for matching sequences regardless of predicted binding allele. This compensated for the limited allele predictive power of MHC-NP. Exact matches between the data sets as well as near matches—those MHC-NP hits that were 1 residue off from the class I starting position or predicted sequence length (9-mer vs 10-mer)—were noted (Figs 1c and 2c).

### Population Coverage

Population coverage was determined for promising peptides using the IEDB population coverage tool^43^. For those peptides that were non-MHC class II predicted binders, only the MHC class I setting was used. The combined MHC class I and II setting was used for peptides predicted to bind both MHC class I and II. Population coverage was calculated for the world population as well as South America. Restricted alleles came from the IEDB MHC class I and II predictive binding tools. For all class II alleles, only the beta chains were used in calculating frequency as a proxy of the alpha/beta combined allele in the IEDB reference set given that alpha chains are not considered to contribute significantly towards the binding.

### Immunogenicity Tool

MHC class I and II binding along with natural processing were used to determine potential antigenicity of peptides. To predict immunogenicity of chosen peptides, the IEDB class I immunogenicity tool was used^23^. This tool examines immunogenicity of MHC class I and epitope complexes placing emphasis on residues 4–6 and their physiochemical properties. Default settings for the tool were employed.

### Protein Basic Local Alignment Search Tool (BLASTp)

Promising ZIKV peptides, those that performed well for multiple parameters, were analyzed in a BLASTp query with default parameters against each individual DENV serotype using all available full-length polyprotein sequences on Virus Variation for each collected from 2010–2016 [ref. 21]. Promising DENV peptides, similarly defined, were analyzed in a BLASTp query with default parameters against all available ZIKV full-length polyprotein sequences from Virus Variation^21^. Serotype specific DENV peptides were also analyzed against serotype separated DENV full-length polyprotein sequences. An E-value of less than 1 was used to define significantly homologous sequences in all instances. As an added measure, all putative peptides were analyzed in a BLASTp query with default parameters against all *Flaviviridae* family member sequences in the BLAST database.

### Docking Modeling Studies

Predictive docking models were generated using PatchDock^44, 45^ to further support predicted antigenicity and immunogenicity in IEDB. Three-dimensional structures of chosen ZIKV and DENV peptides were first generated using the “Natural Peptides Module for Beginners” feature of PEPstrMOD with default settings^46, 47^. PEPstrMOD results were then submitted to PatchDock along with a predicted binding HLA allele using the default settings. HLA alleles were chosen based on availability of high-quality protein crystal structures and predicted population coverage of the allele as demonstrable examples with the greatest possible relevance (1I4F [ref. 48]; 2XPG [ref. 49]; 1XR8 [ref. 50]; 3LKO [ref. 51]). The highest-ranking result for each peptide that showed predicted binding within the HLA binding groove is shown here. All images were rendered using the PyMOL Molecular Graphics System, Version 1.3 Schrödinger, LLC.

## Electronic supplementary material

Supplemental Tables S1-4
