# Supplementary material for: Identification of putative unique immunogenic ZIKV and DENV1-4 peptides for diagnostic cellular based tests
Source: Sci Rep. 2017 Jul 24;7:6218. doi: 10.1038/s41598-017-05980-z (PMC5524841; doi:10.1038/s41598-017-05980-z)
Supplement: Supplementary file 1 — Supplemental Tables S1-4 [file 41598_2017_5980_MOESM1_ESM.pdf]

Identification of putative unique immunogenic ZIKV and DENV1-4 peptides for diagnostic cellular based tests

Aaron L. Oom, Davey Smith, Kevan Akrami

Supplemental Table S1: MHC class I reference allele set

HLA-A*01:01
HLA-A*02:01
HLA-A*02:03
HLA-A*02:06
HLA-A*03:01
HLA-A*11:01
HLA-A*23:01
HLA-A*24:02
HLA-A*26:01
HLA-A*30:01
HLA-A*30:02
HLA-A*31:01
HLA-A*32:01
HLA-A*33:01
HLA-A*68:01
HLA-A*68:02
HLA-B*07:02
HLA-B*08:01
HLA-B*15:01
HLA-B*35:01
HLA-B*40:01
HLA-B*44:02
HLA-B*44:03
HLA-B*51:01
HLA-B*53:01
HLA-B*57:01
HLA-B*58:01

Supplemental Table S2: MHC class II reference allele set

HLA-DRB1*01:01
HLA-DRB1*03:01
HLA-DRB1*04:01
HLA-DRB1*04:05
HLA-DRB1*07:01
HLA-DRB1*08:02
HLA-DRB1*09:01
HLA-DRB1*11:01
HLA-DRB1*12:01
HLA-DRB1*13:02
HLA-DRB1*15:01
HLA-DRB3*01:01
HLA-DRB3*02:02
HLA-DRB4*01:01
HLA-DRB5*01:01
HLA-DQA1*05:01/DQB1*02:01
HLA-DQA1*05:01/DQB1*03:01
HLA-DQA1*03:01/DQB1*03:02
HLA-DQA1*04:01/DQB1*04:02
HLA-DQA1*01:01/DQB1*05:01
HLA-DQA1*01:02/DQB1*06:02
HLA-DPA1*02:01/DPB1*01:01
HLA-DPA1*01:03/DPB1*02:01
HLA-DPA1*01/DPB1*04:01
HLA-DPA1*03:01/DPB1*04:02
HLA-DPA1*02:01/DPB1*05:01
HLA-DPA1*02:01/DPB1*14:01

Supplemental Table S3: ZIKV peptides and predicted HLA binders

**NS1 Residues**	**Sequence**	**Alleles**
16-24/25	KETRCGTGV/KETRCGTGVF	B*40:01, B*44:02, B*44:03
125-133	KSYFVRAAK	A*03:01, A*11:01, A*30:01, A*31:01
158-167	FLVEDHGFGV	A*02:01, A*02:03, A*02:06
166-175	GVFHTSVWLK	A*03:01, A*11:01, A*68:01, DPA1*02:01/DPB1*01:01, DPA1*01:03/DPB1*02:01, DPA1*01/DPB1*04:01, DPA1*03:01/DPB1*04:02
169-177	HTSVWLKVR	A*31:01, A*33:01, A*68:01, DPA1*02:01/DPB1*01:01, DPA1*01:03/DPB1*02:01, DPA1*01/DPB1*04:01, DPA1*03:01/DPB1*04:02

Supplemental Table S4: DENV peptides and predicted HLA binders

Serotype	Residues	Sequence	Predicted Alleles
**1**	31-39/40	MLMTGTLAV/ MLMTGTLAVF	A*02:01, A*02:03, A*02:06, A*23:01, A*24:02, A*32:01, A*68:02, B*08:01, B*15:01, B*35:01, DPA1*01:03/DPB1*02:01, DPA1*01/DPB1*04:01
**2**	33-41	ILLVAVSFV	A*02:01, A*02:03, A*02:06, DPA1*01:03/DPB1*02:01, DPA1*01/DPB1*04:01, DQA1*04:01/DQB1*04:02
**2**	63-72	TMTDDIGMGV	A*02:01, A*02:03, A*02:06
**3**	31-40	HMIAGVFFTF	A*23:01, A*24:02, B*15:01, DPA1*01:03/DPB1*02:01, DPA1*01/DPB1*04:01, DPA1*03:01/DPB1*04:02, DPA1*02:01/DPB1*05:01, DRB1*11:01
**3**	32-40/41	MIAGVFFTF/ MIAGVFFTFV	A*02:01, A*02:03, A*02:06, A*23:01, A*24:02, A*26:01, A*32:01, A*68:02, B*15:01, B*35:01, B*53:01, B*58:01, DPA1*01:03/DPB1*02:01, DPA1*01/DPB1*04:01, DPA1*03:01/DPB1*04:02, DPA1*02:01/DPB1*05:01, DRB1*11:01
**3**	157-165/166	TIFTLTVAW/ TIFTLTVAWR	A*31:01, A*32:01, A*33:01, A*68:01, B*57:01, DRB1*07:01
**4**	81-90	KMSPGYVLGV	A*02:01, A*02:03, A*02:06, DRB1*01:01, DRB1*09:01, DRB3*02:02, DRB5*01:01
**Consensus**	15-23/24	MAIFIEEVM/ MAIFIEEVMR	A*33:01, A*68:01, B*35:01, B*53:01, B*57:01, B*58:01, DRB1*03:01, DRB1*11:01
**Consensus**	42-50/51	LLIMGQLTW/ LLIMGQLTWR	A*31:01, B*53:01, B*58:01, DRB1*11:01, DRB5*01:01
**Consensus**	86-94/95	MFAVGLLLR/ MFAVGLLLRK	A*03:01, A*31:01, A*33:01, A*68:01, DRB1*11:01, DPA1*01/DPB1*04:01, DPA1*03:01/DPB1*04:02
**Consensus**	132-140	MMLKLVTNF	A*23:01, A*32:01, B*15:01, DPA1*02:01/DPB1*05:01, DRB5*01:01
**Consensus**	143-152	YQLWTTLLSL	A*02:01, A*02:06, B*15:01, DPA1*01/DPB1*04:01, DPA1*03:01/DPB1*04:02, DPA1*02:01/DPB1*14:01, DRB1*04:01, DRB1*11:01
**Consensus**	170-178	MVLAVVSLF	A*23:01, A*26:01, B*35:01, B*57:01, B*58:01, DPA1*02:01/DPB1*01:01, DRB1*03:01
